# Hereditary Gingival Fibromatosis: A Report of a Severe Case

**DOI:** 10.7759/cureus.23280

**Published:** 2022-03-17

**Authors:** Samia Aboujaoude, Georges Aoun

**Affiliations:** 1 Pediatric Dentistry and Public Dental Health, Lebanese University, Beirut, LBN; 2 Oral Medicine and Maxillofacial Radiology, Lebanese University, Beirut, LBN

**Keywords:** familial, general anesthesia, hereditary gingival fibromatosis, gingivectomy, gingival overgrowth

## Abstract

Hereditary gingival fibromatosis (HGF) is an uncommon condition characterized by a benign, local, or diffuse gingival overgrowth. It may cover the teeth partially or totally, causing essential aesthetic, phonetic, and masticatory disorders. In this report, we discuss a case of an 11-year-old boy who presented with severe gingival enlargement. The diagnosis of HGF was made based on clinical examination and family history, with two of the patient’s brothers and his paternal aunt being affected with the same disease. The patient was managed with electrosurgery under general anesthesia.

## Introduction

Gingival fibromatosis (GF) is a rare condition characterized by a benign, local, or diffuse gingival overgrowth [1,2]. It involves the maxilla and the mandible and can affect both sexes [1,3]. Hereditary gingival fibromatosis (HGF) is defined as GF linked to hereditary factors and may occur as a non-syndromic entity or coexist with many genetic syndromes [1] such as infantile systemic hyalinosis [4], juvenile hyaline fibromatosis [5], and Rutherfurd syndrome [6]. In HGF, the gingiva appears firm and pink without any sign of inflammation [3]. It covers the teeth totally or in part, leading to significant aesthetic, phonetic, and masticatory disorders. The overgrowth involves the attached gingiva, the gingival margin, and the interdental papillae [2,7].

The main differential diagnosis of HGF is gingival hyperplasia induced by drugs (GHID) such as phenytoin, cyclosporine, and nifedipine [3,8]. Unlike HGF, which is characterized by slow, progressive growth of the gingival tissue, GHID habitually occurs as a generalized enlargement many months after the onset of systemic therapy. Clinically, GHID may be accompanied by a secondary inflammation due to the presence of pseudopockets and plaque accumulation; the gingiva becomes erythematous and appears smooth, and red or bluish-red. Furthermore, periodontal problems such as bleeding and bone loss might also occur [1,8]. The rate of prevalence of HGF is unknown, but many cases may be found within the same family [3]. The etiopathogenesis of HGF remains unidentified although many researchers have observed an increase in sub-epithelial fibroblast proliferation and collagen and fibronectin synthesis with a decrease in the matrix metalloproteinases [3,9].

Various treatment procedures for HGF have been reported, mainly the surgical and laser-assisted excision of gingival enlargement [1]. In this report, we describe a case of an 11-year-old boy with severe HGF as well as the treatment adopted.

## Case presentation

**Figure 1:**
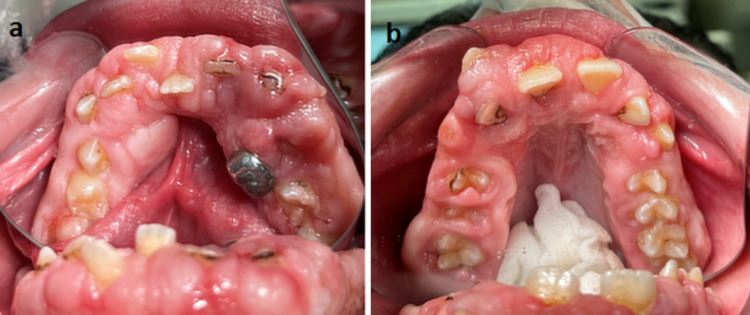
Intraoral photographs - 1 Intraoral occlusal photographs showing the severe gingival overgrowth involving both the mandible (a) and the maxilla (b)

An 11-year-old boy presented with his parents to the Department of Pediatric Dentistry at the Lebanese University complaining of swollen appearance of the gingiva causing bad aesthetic appearance and masticatory and phonation difficulties (Figure 1).

**Figure 2:**
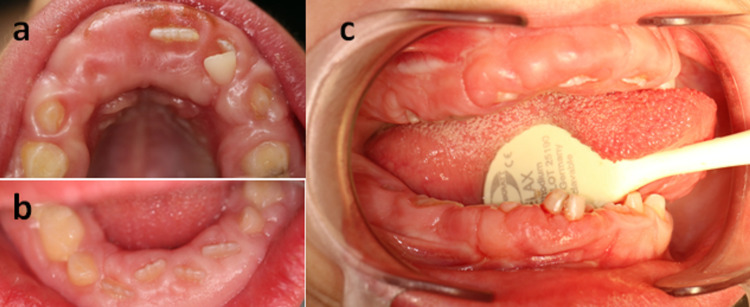
Intraoral photographs of the patient's brothers Intraoral photographs showing the gingival overgrowth involving both the mandible and the maxilla of the first brother aged nine years (a, b) and the second brother aged seven years (c)

A thorough history revealed that the patient had two brothers with HGF as well as a paternal aunt who had been treated for the same condition. No medication intake known to cause gingival overgrowth was reported. All three brothers were mentally retarded and deaf-mutes and suffered from open bite and lips incompetence. The intraoral examination of the patient's brothers also revealed severe gingival enlargement in both the mandibular and maxillary arches (Figure 2).

Radiological examination showed no periodontal involvement in any of the three children. Given the patient's age and medical situation, we discussed the treatment plan with the parents, which would consist of excising the fibrotic gingival tissue under general anesthesia, and they provided their written consent.

**Figure 3:**
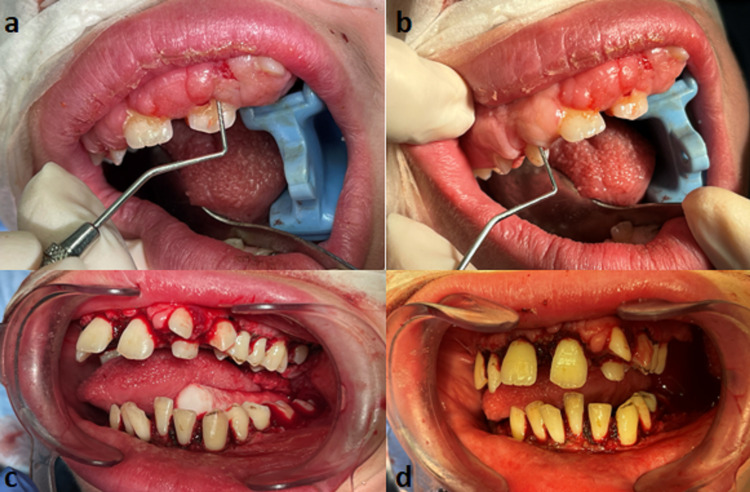
Intraoral photographs - 2 Intraoral photographs showing the steps of the surgical technique from the probing measurement before the procedure (a, b) to the outcomes immediately after the gingivectomy (c, d)

In the operating room after induction, nasal intubation and pharyngeal pack placement were achieved. Intraoral periapical radiographs were taken with a portable X-ray; dental prophylaxis and preventive and restorative treatments such as preventive resin and composite restorations were performed. To achieve hemostasis, we completed the gingivectomy using an electrosurgical device (ERBE VIO 3, Erbe Elektromedizin GmbH, Tübingen, Germany) in monopolar cutting/coagulation mode with an incising electrode. We began by measuring the overgrowth with a periodontal probe and cautiously used the cautery tool to make delicate changes to the gingival marginal level to eliminate gingival excess. Careful technical considerations were followed to respect the biological width and not to exceed 3 mm of the base of the pocket (Figure 3).

The patient was discharged from the hospital on the same day with a prescription of non-steroidal anti-inflammatory drugs (Ibuprofen Profinal® Pediatric Suspension, Julphar 5 ml Suspension) every six hours, to reduce pain and possible swelling following the treatment, for three days. A prophylactic antibiotherapy (Ospamox® 250 mg/5 ml, 25 mg/kg/day) was recommended for seven days to prevent post-procedural infections following the invasive electrosurgery. The day after the surgery, wet gauze embedded with chlorhexidine gluconate 0.12% (GUM, Paroex®) was recommended to wipe the teeth and gums for six days since the child was not capable of maintaining proper hygiene. The parents were advised to start feeding him a liquid diet.

**Figure 4:**
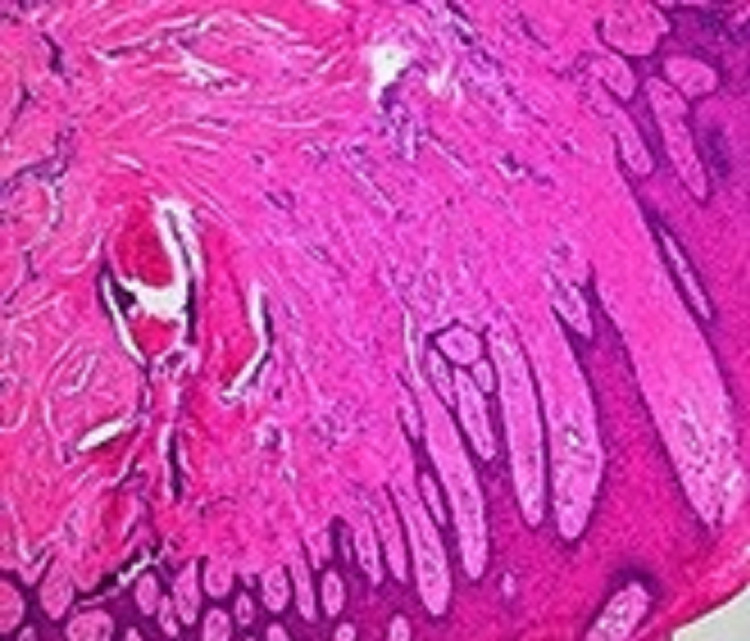
Histological section Histological section showing hyperplastic overlying epithelium with elongated epithelial ridges that project deeply into the underlying connective tissue

Histological examination of the specimen showed hyperplastic overlying epithelium with elongated epithelial ridges that projected deeply into the underlying connective tissue, the latter being moderately dense with collagen bundles randomly arranged (Figure 4).

**Figure 5:**
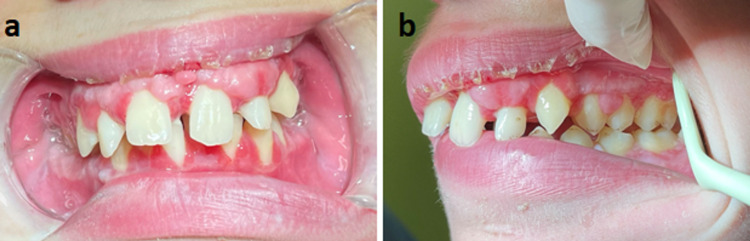
Intraoral photographs - 3 Photographs after 10 days from the surgery showing the results anteriorly (a) and laterally (b)

A routine follow-up examination was carried out after 10 days (Figure 5) and parents were given dietary counseling as well as oral hygiene instructions.

## Discussion

HGF is a highly unusual condition characterized by a benign gingival overgrowth. It is inherited in an autosomal-dominant or, less frequently, autosomal-recessive manner [10,11]. Autosomal-dominant non-syndromic forms of HGF acquired by inheritance are associated with the chromosomes 2p21-p22 and 5q13-q22 [11]. Lately, a mutation in the gene Son of Sevenless-1 (SOS-1) has been linked to this condition [10-12].

Usually, the overgrowth starts with the appearance of primary dentition, and it progressively extends to totally cover the teeth [10]. Severe cases result in crowding of underlying teeth, phonation disturbance, and disorders in mastication [12]. The enlarged gingiva generally presents normal color and consistency with nodular and stippled gingiva, and these aspects were seen in our case.

According to many authors, the management of HGF depends on the severity of the gingival overgrowth [1,12]. The classic treatment is surgical with a scalpel, mainly external bevel gingivectomy, followed by the use of 0.12% chlorhexidine mouthwash for a couple of weeks. Other practitioners have recommended that the excision of the enlarged tissue can be performed by electrosurgery or by laser (Table 1); these two techniques help in reducing operative pain and hemorrhage and restore normal gingival appearance and contours [1,13].

**Table 1:** Types of treatment for gingival overgrowth in some case reports in the literature HGF: hereditary gingival fibromatosis; IDF: idiopathic gingival fibromatosis

Studies	Patient gender	Patient age (years)	Type of gingival overgrowth	Management
Mohan et al. [7]	Male	22	HGF	Surgical gingivectomy
Sharma et al. [2]	Female	8	HGF	Two-stage gingivectomy
Almiñana-Pastor et al. [3]	Male	8	HGF	Internal bevel gingivectomy
Almiñana-Pastor et al. [3]	Male	8	HGF	External bevel gingivectomy
Anand Nayak et al. [10]	Female	11	IGF	External bevel gingivectomy
Jaju et al. [11]	Female	18	IGF	External bevel gingivectomy
Ramakrishnan et al. [12]	Male	16	HGF	External bevel gingivectomy
Gontiya et al. [13]	Female	13	IGF	Laser-assisted gingivectomy
Present case	Male	11	HGF	Electrosurgery

It is to be noted that irrespective of the surgical technique used, long-term postoperative observation is necessary due to the high-recurrence rate [10]. In our case, electrosurgery was employed with regular postoperative follow-up visits.

## Conclusions

When encountering localized or diffuse gingival overgrowth, a thorough medical and familial history coupled with meticulous clinical and radiological assessments are mandatory in order to investigate the presence of HGF. Despite the benign nature of this condition, a suitable treatment must be chosen with a long follow-up period to avoid probable recurrence. Electrosurgery is a very effective technique to correct the gingival appearance and other disturbed functions.
